# Assessment of the Temporal Trajectory of Clinical Trials for COVID-19 Interventions After Highly Publicized Lay and Medical Attention

**DOI:** 10.1001/jamanetworkopen.2021.0689

**Published:** 2021-03-04

**Authors:** Nadir Yehya

**Affiliations:** 1 Department of Anesthesiology and Critical Care Medicine, Children’s Hospital of Philadelphia, University of Pennsylvania, Philadelphia

## Abstract

This cross-sectional study assesses the temporal trajectory of clinical trial registration for COVID-19 interventions that were highly publicized during the COVID-19 pandemic vs treatments that were not comparably promoted.

## Introduction

The coronavirus disease 2019 (COVID-19) pandemic has triggered an explosion of human subjects research in attempts to stay ahead of the virus. Clinical research is neither free in terms of cost nor without patient risks—particularly interventional trials, which remain the most common design for testing efficacy. With some exceptions, patients enrolled in a trial are typically ineligible for competing trials for the same condition that assess a different intervention. Because COVID-19 was increasingly politicized, there was some concern about the undue influence of partisans on registration of drug trials, specifically for hydroxychloroquine. Primarily on the basis of a single case series,^1^ hydroxychloroquine was subject to exaggerated attention in the lay and medical press, including social media.

## Methods

A cross-sectional study was performed to compare the temporal trajectory of registered trials for hydroxychloroquine with that for corticosteroids, similarly inexpensive drugs with promising preliminary data that were not comparably promoted. This study was reviewed by the Children’s Hospital of Philadelphia Institutional Review Board and was deemed exempt from further review because it was an analysis of registered trials. Per the Strengthening the Reporting of Observational Studies in Epidemiology (STROBE) reporting guideline, trials registered at ClinicalTrials.gov between February 1 and September 20, 2020, were assessed to examine growth in the number of interventional trials of either hydroxychloroquine or any corticosteroid for treatment of COVID-19. Given concern that exaggerated attention inappropriately influencing trial registration was not limited to COVID-19, a separate assessment of sepsis trials posted between January 1 and September 20, 2020, was conducted to compare the growth in the number of interventional trials of a drug that received exaggerated attention—vitamin C^2^—with 2 similarly inexpensive drugs that did not receive such attention (corticosteroids and heparin). Statistical analyses were performed with SigmaPlot 13 (Systat Software).

## Results

**Figure 1.**
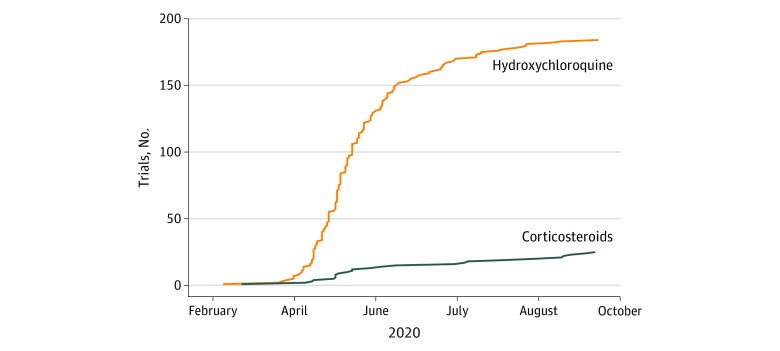
Number of Registered Interventional Trials for Medications for Coronavirus Disease 2019 (COVID-19) in ClinicalTrials.gov Exponential growth in the number of registered clinical trials of hydroxychloroquine as an intervention for COVID-19 occurred in March 2020 after the publication and promotion of key preliminary studies, in contrast with trials of corticosteroids.

Of the trials for COVID-19 interventions that were highly publicized, 184 trials were for hydroxychloroquine and 25 for corticosteroids (Figure 1). Trial registrations for hydroxychloroquine grew exponentially starting in late March 2020, with the US Food and Drug Administration providing emergency use authorization for hydroxychloroquine for COVID-19 on March 28, 2020 (which was subsequently revoked). Trial registrations increased nearly 6-fold from 0.3 trials registered per day between February 1 and March 28, 2020, to 1.7 trials registered per day between March 29 and June 30, 2020. Trial registrations slowed by August 2020 after negative findings from higher-quality trials.^3^ By contrast, growth in corticosteroid trial registrations was slower and linear, not exponential, increasing slightly from 0.09 trials registered per day to 0.15 trials registered per day before and after March 28, 2020.

**Figure 2.**
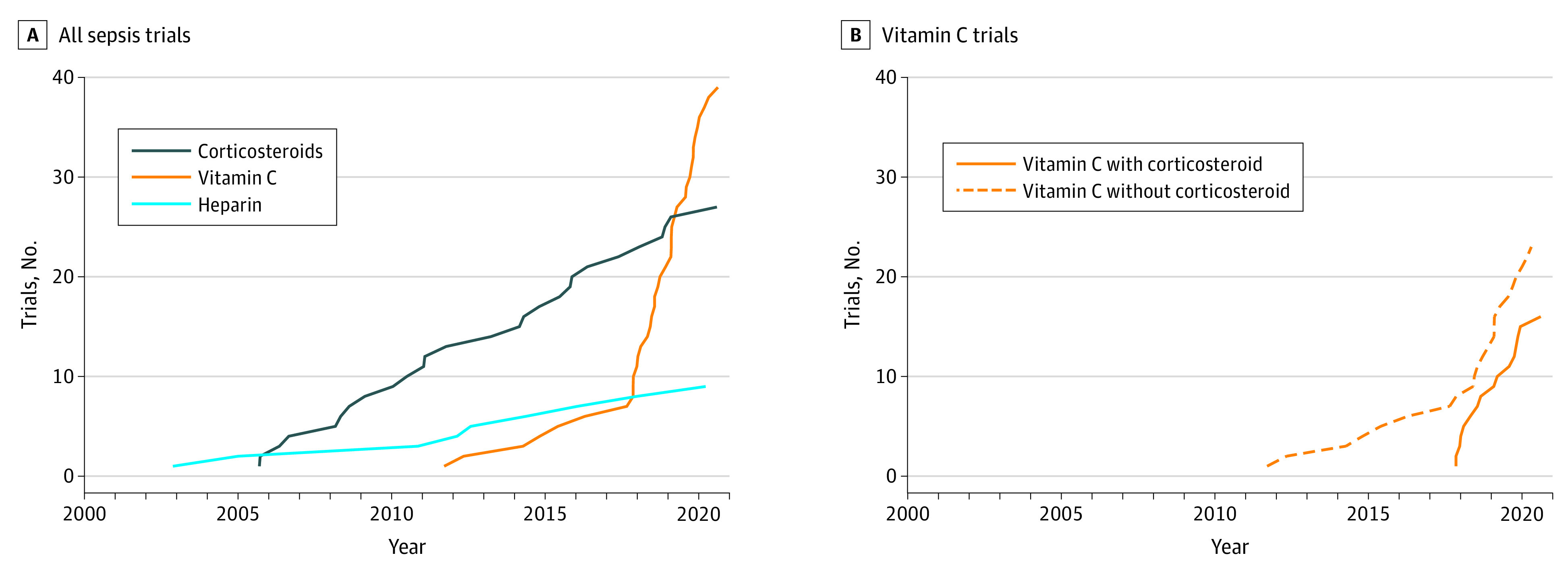
Number of Registered Interventional Trials for Sepsis Treatments A, Exponential growth in the number of trials of vitamin C registered in ClinicalTrials.gov occurred after the publication and promotion of a single-center retrospective cohort study using historical controls, in contrast to trials of corticosteroids and heparin, which showed slow linear increases. B, This exponential growth occurred in all trials with vitamin C, irrespective of whether vitamin C was combined with corticosteroids.

A similar phenomenon was observed in sepsis trials (Figure 2), with 39 trials for vitamin C (16 of which coadministered corticosteroids), 27 for corticosteroids, and 9 for heparin. The number of vitamin C trials showed a sudden rapid increase after publication of a single retrospective study reporting historical controls^2^: 33 of 39 vitamin C trials were registered after 2017. This rapid increase in vitamin C trials occurred irrespective of whether the concurrent use of corticosteroids was tested.

## Discussion

The exponential growth in trials for hydroxychloroquine and vitamin C stands in stark contrast to the slower increase in trials for corticosteroids and heparin. Importantly, this is not a suggestion that hydroxychloroquine and vitamin C represented irrational targets for intervention for COVID-19 or sepsis. Indeed, a single trial of vitamin C suggested mortality benefits in septic acute respiratory distress syndrome.^4^ Of note, however, is that the evidence for use of corticosteroids^5,6^ is superior to that for either hydroxychloroquine or vitamin C for their respective diagnoses. Rather, this study’s findings suggest that the trajectories of trial registrations for hydroxychloroquine and vitamin C were inappropriate and represent the susceptibility of medical fields to intensive publicity and promotion. In both cases, a single small, nonrandomized report accompanied by persistent promotion in the lay press and on social media resulted in a sharp increase in trial registrations and an unearned consumption of resources.

Although the example of hydroxychloroquine was undoubtedly exaggerated by inappropriate politicization and the difficulties of providing midpandemic patient care, the vitamin C example suggests that these errors in judgment also occur absent overt political pressure. Limitations of this analysis include a lack of assessment of trial funding, quality, the likelihood of completion, and pretrial efficacy data. Future investigators should consider these cautionary examples when considering whether preclinical and observational data are sufficiently compelling to embark on large-scale randomized trials.
